# Cannabinoids in Glaucoma Patients: The Never-Ending Story

**DOI:** 10.3390/jcm9123978

**Published:** 2020-12-08

**Authors:** Andrea Passani, Chiara Posarelli, Angela Tindara Sframeli, Laura Perciballi, Marco Pellegrini, Gianluca Guidi, Michele Figus

**Affiliations:** 1 Ophthalmology, Department of Surgical, Medical and Molecular Pathology and Critical Care Medicine, University of Pisa, 56126 Pisa, Italy; andreapassani@gmail.com (A.P.); angelasframeli@gmail.com (A.T.S.); lauraperci1@gmail.com (L.P.); gianlu.guidi@gmail.com (G.G.); michele.figus@unipi.it (M.F.); 2 Ophthalmology, Head and Neck Department, S.Orsola-Malpighi Teaching Hospital-University of Bologna, 40138 Bologna, Italy; marco.pellegrini@hotmail.it

**Keywords:** cannabinoids, glaucoma, ocular hypertension, intraocular pressure, marijuana, cannabis

## Abstract

Glaucoma is one of the principal causes of irreversible blindness worldwide. Yet, intraocular pressure (IOP) is the main modifiable risk factor for disease progression. In the never-ending challenge to develop new and effective drugs, several molecules have been tested as anti-glaucoma agents thanks to their pressure-lowering capabilities. Among these molecules, the cannabinoids have been investigated as possible anti-glaucoma drugs since the early 1970s. Cannabinoids are a large class of chemical compounds that exploit their effects by interaction with cannabinoid receptors 1 and 2. These receptors are widely expressed in the human retina where they may influence important functions such as photo-transduction, amacrine cell network maintenance, and IOP regulation. Therefore, in past years several studies have been conducted in order to assess the IOP lowering effects of cannabinoids. PRISMA guidelines have been used to perform a literature search on Pubmed and Scopus aiming to investigate the mechanism of IOP lowering effects and the potential benefits of orally administered, inhaled, topical, and intravenous cannabinoids in the treatment of glaucoma patients.

## 1. Introduction

Glaucoma is one of the leading causes of irreversible blindness worldwide [1,2]. Intraocular pressure (IOP) is the main modifiable risk factor for preventing disease progression [3]. Several anti-glaucoma agents have therefore been developed in order to lower intraocular pressure and to improve patients’ compliance and quality of life. In the never-ending challenge to develop new and efficient drugs, several molecules have been tested as anti-glaucoma agents thanks to their pressure-lowering capabilities. Among these molecules, the cannabinoids (CB) have been investigated as possible anti-glaucoma drugs since the early 70s [4]. Cannabinoids are a large class of chemical compounds derived from the trichomes and the leaves of *Cannabis* plants (phytocannabinoids) or artificially produced by pharmacological synthesis (synthetic cannabinoids) [5]. These molecules react with cannabinoid receptor 1 (CB1) and cannabinoid receptor 2 (CB2) that are the natural receptors of endocannabinoids, a class of lipid-based neurotransmitters that modulates several physiological and cognitive processes such as appetite, pain sensation and memory [6,7]. Since CB1 and CB2 are expressed in human retina, ciliary body and retinal pigment epithelium, the administration of exogenous cannabinoids may modulate several retinal processes such as signal transduction, photo-transduction and IOP control [8,9,10,11]. For these reasons, cannabinoids have been widely investigated as IOP lowering medications [12,13]. Beside IOP lowering capabilities, cannabinoids show important neuroprotective effects on both central and peripheric nervous system [14,15,16,17]. Cannabinoids’ ability to reduce glutamate release and oxidative stress are indeed well documented [15,17]. Neurodegeneration plays a main role in glaucoma pathogenesis and progression therefore cannabinoids may represent a useful tool thanks to their dual therapeutic effect [18,19]. Obtaining a significant IOP reduction and an efficient neuroprotective effect with a single drug is indeed alluring. For these reasons scientific interest on this class of molecules has arisen in past years. The legalization of medical and recreational use of cannabinoids has otherwise appealed general public attention and curiosity on this topic. For these reasons we think a comprehensive review of pharmacological and therapeutical properties of cannabinoids may be actual and interesting. In this manuscript we will therefore systematically review the mechanism of IOP lowering effects and the potential benefits of orally administered, inhaled, topical and intravenous cannabinoids in the treatment of glaucoma patients.

### 1.1. Phytocannabinoids and Endocannabinoids: Mechanism of Action

**Figure 1:**
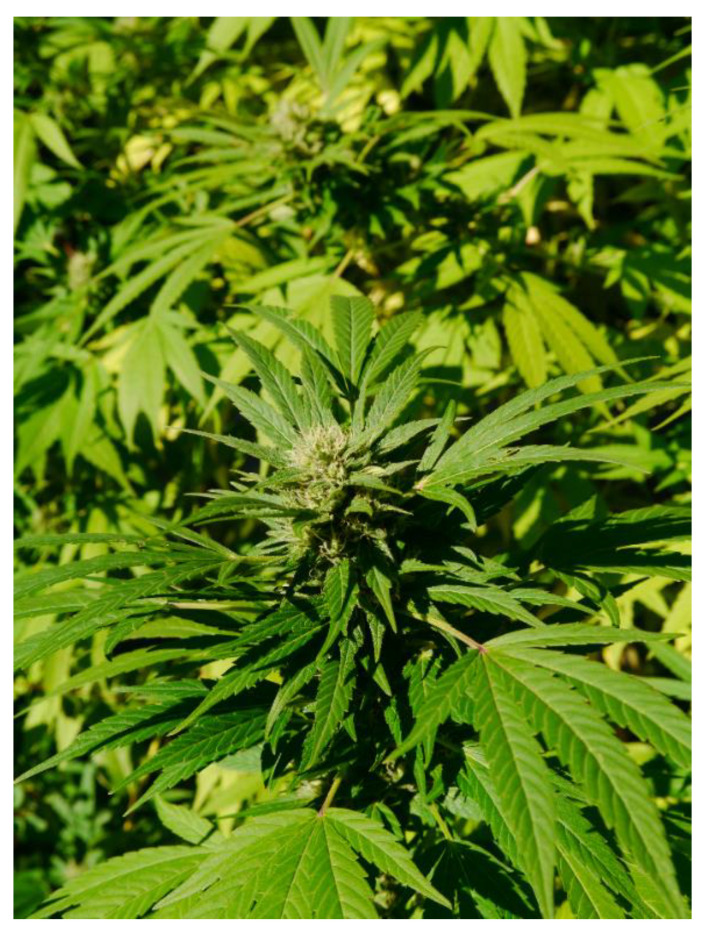
Cannabis sativa plant.

Phytocannabinoids are derived from flowers and leaves of *Cannabis sativa, Cannabis indica, and Cannabis ruderalis*. About 113 different cannabinoids can be isolated from *Cannabis sativa* (Figure 1); the most represented and studied ones are tetrahydrocannabinol (THC), cannabidiol (CBD), and cannabinol (CBN), which present important psychoactive effects [20]. CBD is most abundant in *Cannabis sativa*, THC derives mainly from *Cannabis indica*, while *Cannabis ruderalis* presents low amounts of psychoactive cannabinoids [21,22]. All the aforementioned molecules are synthetized in glandular trichomes of *Cannabis* plants from fatty acid and isoprenoid precursors [23,24]. All cannabinoids consist of a lipid backbone with the incorporation of alkylresorcinol and monoterpene groups with highly lipophilic properties [21,22]. Cannabinoids can be administered orally, topically, or through inhalation. Bioavailability for phytocannabinoids in humans is higher when administered through inhalation: 10–35% for THC, 11–45% for CBD, and 38% for CBN [7,25,26,27]. An extensive first-pass liver metabolism otherwise reduces the oral bioavailability to 5–20% for THC and 13–19% for CBD [28]. No clinical data are available on bioavailability after topical administration in humans; in rabbits, topical THC showed a variable systemic bioavailability between 6 to 40% [29]. Cannabinoids exploit their effects through interaction with cannabinoid receptor 1 (CB1) and cannabinoid receptor 2 (CB2) [30,31]. These G-protein-coupled receptors share 48% of their amino acid sequence identity and their activation causes inhibition of adenylate cyclase with consequent reduced conversion of ATP to cAMP. CB1 receptors are mainly found in the central nervous system (CNS), while CB2 receptors are mainly expressed in peripheral tissues and the immune system. Both receptors find in endocannabinoids their natural ligand.

THC acts as an agonist on CB1 and CB2 receptors and displays psychoactive effects both on the brain and peripheral tissues. CBD has a lower affinity for CB1 but can act as an antagonist on CB1 receptors and consequently modulate psychotropic and other effects of THC. CBD also shows an indirect agonistic action by increasing CB1 receptors’ constitutional activity (“endocannabinoid tone”) and has sedating, antiepileptic, antiemetic, anti-inflammatory, anxiolytic, and neuroprotective properties [32]. CBN is the non-enzymatic oxidation byproduct of THC, it doesn’t show psychotropic properties but exerts sedative, analgesic, anti-inflammatory, and neuroprotective effects [33].

Endocannabinoids are eicosanoid neurotransmitters that modulate several physiological and cognitive processes such as food intake, adipogenesis, and glucose metabolism. Anandamide (N-arachidonoylethanolamine, AEA), Palmitoylethanolamide (PEA) and 2-arachido-noylglycerol (2-AG) are the most studied endogenous cannabinoids as they seem to play an important role in several pathologies such as Parkinson’s and Alzheimer’s disease [21]. Endocannabinoids also play a notable role in eye physiology since 2-AG and PEA are present at high levels the human retina [19,34]. CB1 is expressed in many components of the human retina such as cones, bipolar cells, ganglion cell axons, and amacrine cells, while CB2 is mainly expressed in retinal pigment epithelium (RPE) [19,30,34,35,36]. Activation of retinal endocannabinoid receptors results in ion channel modulation that leads to the release of dopamine, glutamate, and noradrenaline [7,8]. In addition, these receptors modulate the photo-transduction cascade and maintain the amacrine cell network [12]. Some clinical studies demonstrated low concentrations of 2-AG and PEA in glaucomatous eyes [9,19]. These important findings suggest a principal role of the endocannabinoid system in IOP regulation in the human eye.

### 1.2. Cannabinoids Effects on Intraocular Pressure

**Figure 2:**
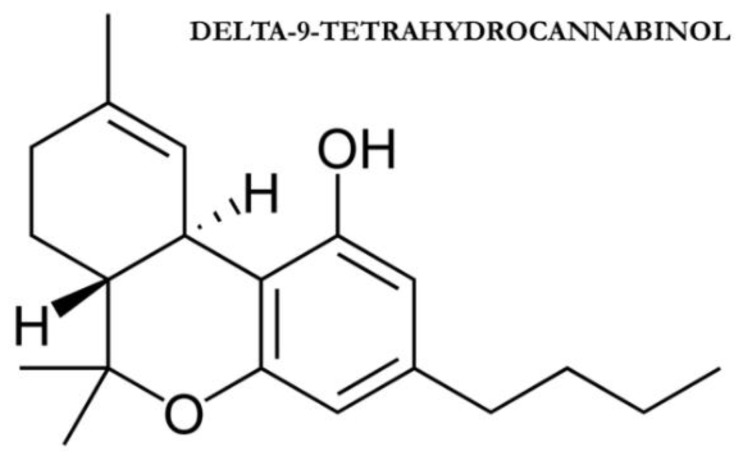
*trans*-delta-9-tetrahydrocannabinol.

Cannabinoids may play an important role in IOP regulation via interaction with the ciliary muscle and Schlemm’s canal, as well as via modulation of cyclooxygenase-2 (COX-2) [37]. All these effects are obtained through interaction with CB1 receptor, as well as by modulation of prostanoids synthesis via cyclooxygenase (COX) pathway [12]. CB1 is widely expressed in both retina and anterior eye structures such as trabecular meshwork, Schlemm’s canal, iris, ciliary body muscle, and ciliary pigmented epithelium. This ubiquitous distribution suggests that multiple pathways may be involved in the IOP lowering effect of cannabinoids through the regulation of aqueous humor production and outflow (trabecular and uveoscleral) [12]. AEA and the synthetic cannabinoid CP 55,940 in fact interact with the CB1 receptor determining ciliary muscle contraction and consequent IOP reduction [38]. Activation of the CB1 receptor in the ciliary muscle may also determine vasodilatation with consequent reduction of aqueous humor production [39]. While AEA and CP55,940 act on the ciliary muscle THC isomer called *trans*-delta-9-tetrahydrocannabinol (delta-9-THC, Figure 2) and cannabigerol determines Schlemm’s canal dilation and consequent outflow facilitation [40]. Both AEA and delta-9-THC also enhance COX-2 expression with consequent increased production of prostaglandin E2 (PGE2) and metalloproteinases 1, 3, and 9 [41]. The enhanced transduction of these molecules determines extracellular matrix remodeling with consequent IOP lowering [41]. While the above-mentioned effects of cannabinoids have been demonstrated by several clinical studies [37,38,39,40,41], the exact role of these molecules in the physiological regulation of IOP is still unclear and needs to be clarified through further clinical trials.

### 1.3. Cannabinoids’ Neuroprotective Effects

Another interesting aspect of cannabinoid usage in glaucoma is connected with the neuroprotective capabilities of these molecules. To date, there are few studies assessing neuroprotective effects of cannabinoids in humans in term of functional or structural changes, but many studies demonstrated better outcomes for retinal ganglion cell (RGC) after different types of injuries in animals (Table 1) [42,43,44,45,46,47,48,49,50,51].

**Figure 3:**
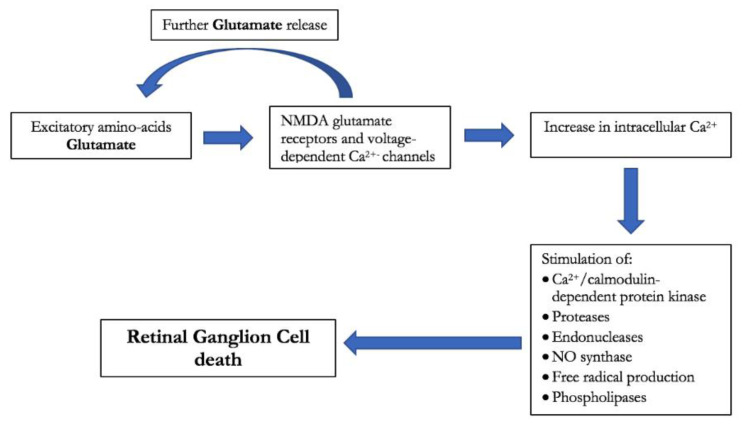
Diagram of glutamate-induced toxicity on retinal ganglion cell.

Three main pathways seemed involved in cannabinoids’ neuroprotective effects: inhibition of glutamate, endothelin-1, and nitric oxide release. Activation of pre-synaptic CB receptors in fact inhibits glutamate release leading to better neuronal excitability and synaptic plasticity [17]. Glutamate is known to increase RGC death via activation of nitric oxide synthase and the consequent increase in oxidative damage. The role of glutamate in glaucoma pathophysiology is well documented [18] (Figure 3). Prolonged administration of delta-9-THC in rats demonstrated a reduction in IOP and a lower RGC death rate by 75% [19]. Cannabinoids’ neuroprotective effects may also be related to their vasodilatation capabilities. The vasoconstrictor Endothelin-1 is higher in patients affected by normal tension glaucoma than in normal controls and may therefore contribute to disease progression [14]. Endogenous cannabinoids may inhibit Endothelin-1 and consequently could play a neuroprotective role thanks to a better optic nerve head blood supply [14]. Oral THC indeed demonstrated to increase retinal perfusion and optic nerve head blood flow even at low dosages [43,44]. THC may therefore represent a promising therapeutic strategy since the optic nerve’s hypoperfusion has been identified as an important risk factor for glaucoma development and progression [52]. The anti-inflammatory effects of cannabinoids may also play a role in their neuroprotective activity. Activation of CB1 and CB2 receptors in the retina and central nervous system inhibits the production of nitric oxide and inflammatory cytokines that are responsible for oxidative stress and RGC death [15]. Oxidative stress reduction may also be obtained by reactive oxygen species (ROS) blockage without any CB receptor activation [16]. In spite of these protective capabilities, some studies otherwise suggest that cannabinoids may be harmful to retinal and nervous cells. A recent study by Schwitzer et al. demonstrated that regular cannabis consumers present a delay in cones’ signal transmission from the central to the near peripheral retina that may determine alterations in precise and color vision [53]. A flash electroretinogram study by Lucas et al. demonstrated an increased retinal background noise in regular cannabis users that may reflect cannabinoids neurotoxicity on retina [54]. Besides long-term effects, cannabis usage may determine early impairment of visual function as suggested by Schwitzer et al. who demonstrated a transient 48% decrease in the a-wave amplitude 30 min after marijuana smoking [55]. These conflictual findings suggest that cannabinoids effects on ocular structures and visual function are heterogeneous and still poorly understood.

## 2. Results

### 2.1. Oral Cannabinoids in Glaucoma and Their IOP Lowering Effects

Several studies have addressed the role of oral cannabinoid administration in glaucoma and are listed in Table 2 [42,56,57,58,59,60,61,62,63,64].

**Figure 4:**
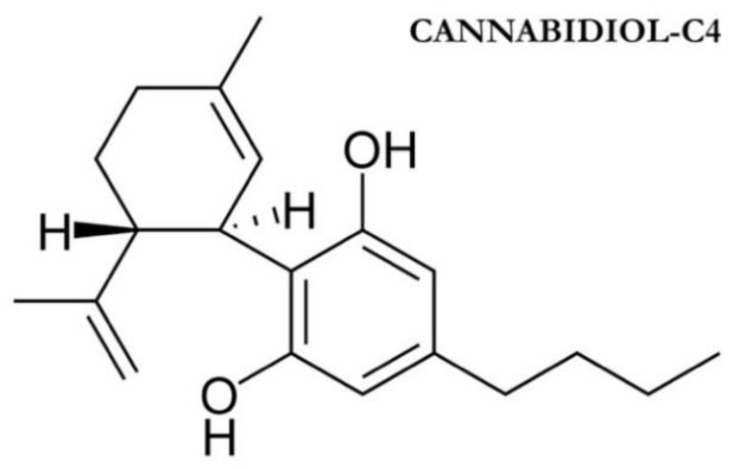
Cannabidiol molecular structure with placebo.

The first case series investigating the effects of oral cannabinoids on IOP both on healthy and glaucomatous patients are the ones published by Hepler in 1976 [56,57]. IOP reduction after 30 min from oral administration of 5, 10, or 20 mg delta-9-THC ranged from 10% to 24% depending on the dosage [56,57]. Subgroup analysis as well as the population studied is described in Table 2. Twenty years apart, Flach investigated long-time IOP lowering effects of orally administered delta-9-THC in 9 end-stage glaucoma patients [60]. In addition to their regular glaucoma therapy, patients were administered from 2.5 to 20 mg oral delta-9-THC four times a day. Dosage was based on clinical indications. The treatment was continued for a maximum of 9 months but most of the patients discontinued the treatment after 8 to 20 weeks due to toxicity phenomena. A transient IOP lowering was noted in all patients but was time-limited due to tolerance development and led to subsequent increases in dosage. This phenomenon was noted in all 9 patients after a few weeks of treatment and resulted in study discontinuation. Only one patient completed the 36-weeks treatment period while the other 8 discontinued the study after 3 to 28 weeks of treatment. The mean IOP reduction was not disclosed by the authors since the patients’ clinical condition, as well as the actual choice of the therapeutic agent, dosage, and duration, were very heterogeneous. The most frequent side effects were dizziness, confusion, sleepiness, anxiety, and depression. The authors’ conclusion was that oral delta-9-THC “can lower intraocular pressure but the eventual development of tolerance and significant systemic toxicity appears to limit the usefulness of this potential treatment” [60]. Subsequently, Tomida et al. in 2006 investigated the IOP lowering capabilities of sublingual administration of delta-9-THC and cannabidiol (CBD, Figure 4) [61]. This pilot study included 6 subjects suffering ocular hypertension or POAG with mild visual field defect (untreated IOP > 24 mmHg). After a 4- to 6-week washout period subjects were administered 5 mg delta-9-THC, 20 mg CBD, 40 mg CBD, or placebo during 4 study visits each taking place one week apart. Delta-9-THC showed a statistically significant reduction of IOP 2 h after administration in comparison.

CBD administration didn’t show any significant additional effect on IOP. Side effects were minimal and limited to nausea and moderate hypotension. The IOP lowering effect of delta-9-THC was modest, temporally limited, and was not considered clinically relevant by the authors [61].

The IOP lowering capabilities of some synthetic derivatives of delta-tetrahydrocannabinol have been investigated by the studies of Tiedman [59], Plange [42], and Newell [58]. Tiedman in 1981 described the hypotensive capabilities of two derivatives of delta-1-tetrahydrocannabinol (BW146Y and BW29Y) [59]. BW29Y was administered orally at a dosage of 5 mg (5 subjects) or 10 mg (5 subjects) to patients suffering ocular hypertension (IOP value between 20–30 mmHg). Both subgroups did not show any significant IOP lowering when compared to the placebo group (6 subjects). Side effects were minimal and statistically non-significant. A statistically significant acceleration of perceived time (“underproduction”) was noted in the 10 mg dosage subgroup. BW146Y was orally administered at a dosage of 4 mg (9 subjects), 8 mg (10 subjects), and 12 mg (3 subjects). A significant IOP reduction was noted in the 8 mg subgroup (mean reduction of 6 mmHg) as well as in the 12 mg subgroup (mean reduction of 9 mmHg). IOP lowering started one hour after drug administration and reached a maximum at 4 h after drug administration. Side effects such as nausea, dizziness, constipation, and drop in blood pressure were dose-dependent and led to a syncopal episode in one of the subjects who was administered the 12 mg dosage.

While Tiedman investigated the IOP lowering capabilities of two delta-1-tetrahydrocannabinol derivates, Plange and colleagues concentrated their attention on a derivate of delta-9-THC called Dronabinol (Marinol, United Pharmaceuticals, Chicago, IL, USA) [42]. Dronabinol 7.5 mg was orally administered to 8 healthy subjects and IOP was measured 2 h after administration. Besides IOP, retinal hemodynamic was also assessed through fluorescein angiography. Mean pretreatment IOP was 13.2 ± 1.9 mmHg, and two hours after Dronabinol administration IOP was significantly lowered to 11.8 ± 2.0 mmHg (*p* = 0.038). In addition to the reduction of IOP, a significant decrease in arteriovenous passage time was also noted (*p* = 0.028). Dronabinol effects on heart rate and blood pressure were limited and non-significant. Prolonged effects on IOP and retinal hemodynamics are still to be investigated.

Newell et al. instead investigated the potential IOP lowering effect of a synthesized crystalline benzopyran called Nabilone [58]. In this study, a single dose of 0.5 mg Nabilone was administered orally to 18 patients (14 suffering POAG and 4 suffering ocular hypertension). IOP decreased by 27.9% in all patients; the maximum therapeutic effect took place between 2 and 4 h after administration. Side effects were few and self-limited. The prolonged effects of oral Nabilone were studied in one patient who underwent oral administration of 0.5 mg of Nabilone three times a day for three weeks. At the conclusion, IOP was significantly lowered (from 40 mmHg to 24 mmHg in the right eye and from 40 mmHg to 22 mmHg in the left) with no significant side effects.

Recently, a renewed interest for oral cannabinoid developed towards endogenous cannabinoid such as Palmitoylethanolamide (PEA) in patients affected by glaucoma or ocular hypertension.

The first study to investigate the role of oral PEA in glaucoma is the one from Gagliano et al. in 2011 [62]. In this crossover study 42 patients already treated with timolol 0.5% presented a baseline mean IOP of 21.6 ± 1.7 mmHg. Subjects were randomized to receive oral PEA (300 mg twice a day) or placebo tablets for two months. Thereafter, both groups underwent a washout period of one month. Finally, patients who had received PEA were switched to placebo while the patients in the placebo group were switched to treatment with 300 mg oral PEA (crossover) for further 2 months. After treatment with oral PEA IOP was lowered by 3.2 ± 1.3 mmHg after one month and by 3.5 ± 1.2 mmHg after two months (*p* < 0.001). No changes in vital signs, visual field, or visual acuity were registered. These interesting results may suggest PEA as a valuable tool in the treatment of glaucoma.

The role of oral PEA in lowering IOP was also investigated by Pescosolido et al. in 2011 [63]. In this study 15 unmedicated patients scheduled for bilateral prophylactic iridotomy were administered with placebo tablet twice a day for 15 days prior to the first eye treatment and then switched to 15 days of oral PEA (300 mg twice a day) for 15 days prior to treatment of the opposite eye. IOP was measured at baseline (t-1), two weeks after treatment with placebo or PEA (t0), and 15, 30, and 120 min after laser treatment (t1, t2, and t3). When PEA was administered post-treatment IOP rise was lower than after placebo administration. This finding was significant for all post-treatment IOP measurements (t1, t2, and t3) but was significantly higher at t2 and t3. From these results, the authors concluded that oral PEA was effective in reducing IOP spikes after laser iridotomy.

Finally, Strobbe et al. conducted a double-blind, placebo-controlled, crossover study with 40 subjects affected by ocular hypertension [64]. They were stratified into two groups that received either placebo or 300 mg PEA tablets twice a day for three months. Thereafter, IOP and endothelium-dependent flow-mediated vasodilatation (FMD) were measured. After PEA administration IOP and FMD showed a significant improvement compared to placebo administration. IOP lowering was 3.7% compared to baseline (*p* < 0.001) while FMD improvement was 40% compared to baseline. From these results, authors concluded that PEA administration for a three-month period determines an improvement in FMD function and a limited but statistically significant IOP lowering.

### 2.2. Inhaled Cannabinoids in Glaucoma and Their IOP Lowering Effects

The first study to investigate the IOP lowering effects of smoking marijuana was published by Hepler et al. in 1971 [4]. In this case series of 11 healthy subjects, 2 g of marijuana (with a 0.9% delta-9-THC) was administered to each subject before IOP measurement. IOP decreased by 30% in almost all subjects but tended to rise again after a few hours. Unfortunately, the published data about this study are limited since study protocol and statistical analysis are not available for review. We nevertheless decided to include this article in our review due to its historical value.

In 1975, Flom et al. conducted a double-blind study on 15 healthy subjects who were administered a 0.8 g cigarette containing either placebo or 12 mg delta-9-THC [20]. IOP lowering in the marijuana group was statistically significant 80 min after drug administration (mean reduction of 2.1 mmHg from a mean baseline IOP of 14.6 mmHg) but was not significant at any other post-treatment interval. Interestingly, subjects who experienced the maximum IOP drop were also those who had the highest score at the Subjective Drug Effects Questionnaire (SDEQ) that was administered to all participants. IOP drop was not related to blood pressure or pulse rate. IOP lowering effects of marijuana seemed to be influenced by tolerance; subjects who routinely smoked marijuana tended to experience low or no IOP lowering after drug administration.

A case series by Hepler et al. reported significant IOP reduction after oral or inhaled administration of delta-9-THC [56]. The study included 256 subjects who either received oral 5 to 20 mg of delta-9-THC, or inhaled cigarettes containing 1 to 4% delta-9-THC. IOP lowering after 30 min from administration varied from 14% to 24% for the orally administered groups and from 25% to 34% for the smoked marijuana groups. A similar IOP drop was maintained 180 min after treatment. Unfortunately, IOP measurements were taken only during the first 4 h after treatment and have not been performed in all subjects. The prolonged effects of delta-9-THC on IOP remain therefore unclear. The scientific significance of this work may be limited by its retrospective nature as well as by the poor homogeneity of the patient population.

In the same year (1976) Hepler et al. published another study regarding IOP lowering capabilities of inhaled or ingested delta-9-THC [57]. In this study, 40 healthy subjects were randomized to be administered with natural or synthetic delta-9-THC derivates. Authors described a 30% IOP lowering with both natural and synthetic delta-9-THC while IOP reduction after oral delta-9-THC was between 15% and 20% depending on the dose. Interestingly subjects administered with THC-free marijuana cigarettes experienced a 10% IOP drop demonstrating that compounds other than delta-9-THC may influence IOP.

Merritt et al. studied the effects of marijuana smoking on both intraocular and blood pressure of glaucomatous patients [65]. In their study 18 subjects suffering either POAG or secondary glaucoma (mean IOP 28.2 mmHg) were assigned to smoke one 900 mg cigarette containing 2% delta-9-THC or placebo. Blood and intraocular pressure as well as other systemic parameters were then measured every 30 min for 4 h. In subjects receiving marijuana cigarettes, IOP decreased significantly (4.1 ± 1.5 mmHg) within the first 30 min after drug administration and reached the maximum decrease (6.6 ± 1.5 mmHg) after 90 min. Both systolic and diastolic blood pressure were significantly reduced in patients receiving inhaled delta-9-THC. Blood pressure decrease was maximum within 10 to 15 min after drug administration and resulted in postural hypotension in 5 cases. Interestingly, the greatest decrease in IOP and blood pressure were registered in patients who presented with essential hypertension. This observation may suggest that delta-9-THC effects on IOP are partially determined by hypotensive effects on blood pressure. Side effects such as drowsiness, hunger, and conjunctiva hyperemia were limited; postural hypotension was the most serious complication that occurred. Table 3 resumes the published evidence on inhaled cannabinoids in glaucoma.

### 2.3. Topical Cannabinoids in Glaucoma and Their IOP Lowering Effects

Topical administration of cannabinoids has been studied since the early 80s but still presents some important and limiting issues [51]. Natural cannabinoids are, in fact, very lipophilic therefore they do not dissolve in water-based vehicles that are usually better tolerated by the eye [66]. For this reason, light mineral oil has been the preferred vehicle for topical delivery of cannabinoids in most studies. Nevertheless, light mineral oil presents some important issues such as water solubility and local toxicity. Water solubility is important for ocular permeability since the eye is constantly cleaned and moistened by tear film [12]. Light mineral oil is water-insoluble and this strongly limits its corneal permeability since tears constantly remove insoluble particles eventually present on the ocular surface. Moreover, light mineral oil presents local toxicity that manifests with lid swelling, burning sensations, and conjunctival hyperemia. During the past decades light mineral oil appeared as the easiest vehicle but this choice may have strongly limited ocular penetration and efficacy of tested drugs. New molecules such as cyclodextrins may represent a better vehicle for topical cannabinoids administration since they increase both cannabinoids’ corneal permeability and ocular tolerability [12]. The first studies to investigate IOP-lowering effects of topical delta-9-THC eye solution are the ones from Merritt et al. (1981) [67,68]. In the first article, the authors investigated the potential IOP lowering effects of a 0.05% and 0.1% topical delta-9-THC eye solution in six hypertensive glaucoma subjects [67]. The compound was diluted in light mineral oil and was administered in one eye of each patient while the contralateral eye was used as a control and treated with vehicle alone. Both concentrations failed to reduce significantly IOP when compared with vehicle alone. No effects on blood pressure were registered. Similar results are described in a subsequent study published in 1981 by the same author [68]. In this second study 8 hypertensive glaucoma patients were administered with 0.01%, 0.05%, or 0.1% delta-9-THC eye solution; ocular and blood pressure were then measured. When compared to placebo topical delta-9-THC eye solution didn’t show any significant IOP lowering effect. A limited blood pressure reduction was noted but its clinical significance has not been furtherly investigated [68].

The above-mentioned observations were confirmed by Green et al. in 1982 [69]. One drop of 1% delta-9-THC diluted in light mineral oil or mineral oil alone was administered in one eye of each subject, and the contralateral eye was used as control. Treatment was administered to 16 healthy subjects with a mean baseline IOP of 15.95 mmHg and the IOP was measured 1, 3, and 6 h after eye drop instillation. No statistically significant decrease in IOP was noted in patients receiving topical delta-9-THC. A light bilateral mydriasis was described in subjects exposed to topical delta-9-THC as well as a mild conjunctival injection. Side effects such as burning sensation and tearing were moderate and equally distributed in the treatment and placebo group. These findings suggested that these ocular side effects may be related to a vehicle’s topical toxicity rather than to the drug’s effect even if cannabinoids showed to stimulate vanilloid receptor type 1 (VR_1_) with a consequent irritating effect similar to capsaicin [7].

Long-term effects of 1% delta-9-THC eye solution were investigated in another study by Jay et al. [66]. In this study, an eye drop containing 1% delta-9-THC or placebo was administered four times a day to 28 healthy subjects for one week. Five subjects (4 exposed to placebo and 1 exposed to delta-9-THC) discontinued the study due to ocular side effects such as lid swelling and burning sensation. In the 23 subjects who completed the study no significant difference in IOP values was found between treatment and placebo group or between treated and untreated eyes.

In 2001, Porcella et al. investigated the effects on IOP of a synthetic cannabinoid receptor agonist called WIN55212-2 [70]. The molecule was mixed with 2-hydroxypropyl-β-cyclodextrin to overcome low aqueous solubility and was then diluted with a saline solution in order to obtain two different concentrations (25 µg or 50 µg). Two drops of WIN55212-2 solution (25 µg or 50 µg) were administered in one eye of 8 subjects and IOP was measured every 15 min for three hours after drug administration. In the subjects receiving the WIN55212-2 solution, IOP decreased by 15 ± 0.5% in the 25 µg group and by 23 ± 0.9% in the 50 µg group after 30 min from drug administration. The IOP lowering effect peaked 1 h after administration (−20 ± 0.7% for 25 µg group and −31 ± 0.6% in 50 µg group) and tended to dissipate by 2 h. Interestingly, an IOP lowering was also noted in the eye not receiving the drug even if this decrease was not statistically significant. No major side effects were noted, and the solution presented good stability and tolerability.

A recent case series of 5 patients has been described by Pescosolido et al. in 2018 [71]. In this study, patients were administered topical Bediol (containing 3–6 mg/mL of delta-9-THC and 4–8 mg/mL of cannabidiol) for 30 days. Then, after one month of washout, the patients were then switched to topical Bedrocan (containing 18–23 mg/mL of delta-9-THC and 1.2–18 mg/mL of cannabidiol) for an additional 30 days. IOP didn’t show any significant lowering in 4 out of 5 patients. Only one patient, affected by intractable uveitic glaucoma, showed a significant IOP reduction after treatment with Bedrocan (IOP lowering of 20% from baseline). The IOP decrease was accompanied by a relevant improvement of the inflammatory condition of the eye. This observation suggests that the IOP lowering effect of Bedrocan is strictly linked to its anti-inflammatory capabilities. No major side effects were recorded. All these studies are summarized in Table 4.

Besides the scientific literature, a marijuana-based eye drop called Canasol has been sold in Jamaica and in other Caribbean countries since the early 90s [72]. These eye drops claim to significantly lower IOP but no scientific evidence and no randomized controlled studies are available. Therefore, its efficacy and safety remain unknown.

### 2.4. Intravenous Cannabinoids in Glaucoma and Their IOP Lowering Effects

The first study that documented a significant IOP lowering effect of intravenous delta-9-THC is the one from Purnell and Gregg (1975) [73]. In this study, 3 to 6.7 mg of albumin-solubilized THC was intravenously administered to two healthy subjects. IOP lowering peaked at 60% and returned to baseline values 240 min after drug administration. Significant euphoria, dizziness, and confusion were recorded in both subjects (Table 5).

A randomized study was carried out two years later by Cooler and Gregg and involved 10 healthy subjects [74]. The enrolled subjects were randomized to intravenously receive either 0.022 mg/Kg delta-9-THC, 0.044 mg/Kg delta-9-THC, 0.157 mg/Kg, diazepam sodium, or placebo (human serum albumin). IOP was measured every 30 min for the first 24 h following the injection, as well as blood pressure and heart rate. All subjects receiving intravenous delta-9-THC showed a significant dose-dependent IOP decrease. Subjects administered 0.022 mg/Kg delta-9-THC showed a 29% IOP lowering versus 37% in subjects receiving 0.044 mg/Kg. Patients treated with diazepam sodium experienced a moderate IOP decrease of 10%, as well as those subjects treated with placebo (2%). The IOP lowering effect was maximum between 30 and 90 min after injection with a peak activity duration of less than 90 min. Blood pressure didn’t change after delta-9-THC administration but three subjects (two who received the higher dose and one who received the lower dose) experienced a marked decrease in blood pressure with concomitant pre-syncopal symptoms. No other serious adverse events were reported.

## 3. Discussion

Cannabinoids’ effects on intraocular pressure have been investigated since the early 70s thanks to the works by Hepler and Cooler [4,56,57,74]. These authors demonstrated a significant IOP lowering effect of inhaled, injected, or orally administered cannabinoids. Besides these encouraging results, the interest in cannabinoids as a treatment for glaucoma has recently increased in the scientific community. This renewed interest in cannabinoids can be explained by the discovery of their neuroprotective role and by the recognition of neuronal degeneration as a key factor for glaucoma pathogenesis [75]. Indeed, cannabinoid receptors are represented in the human retina where their activation improves synaptic plasticity through inhibition of glutamate release and inhibits the production of nitric oxide [17]. Cannabinoids also reduce reactive oxygen species, prevent vasoconstriction, and inhibit the production of inflammatory cytokines [15]. These multi-level neuroprotective mechanisms may suggest cannabinoids as an important tool in preventing glaucoma neurodegeneration. Therefore, the possibility of achieving an IOP lowering effect and a neuroprotective action with only one molecule is undoubtfully alluring [12]. Recently, interest in cannabinoids has also risen thanks to the approval of marijuana for medical purposes by several European countries as well as the United States [76,77]. Medical marijuana is used for many therapeutic purposes such as chronic pain control, nausea, and seizure disorders, and is acquiring patients’ interest as an anti-glaucoma treatment too [5,21,76]. The academic world’s renewed interest in cannabinoids is also demonstrated by the recent review by Wang and colleague who described the numerous effects of cannabinoids on different ocular structures [33]. Different from their work, we focused on cannabinoids’ IOP lowering and neuroprotective capabilities and we included in our review only human studies. This choice was made in order to focus on understanding the mechanisms and opportunities connected with cannabinoids’ usage as anti-glaucomaagents. Despite the great interest by both the scientific and general population, the use of cannabinoids as an anti-glaucoma treatment has still far to come. Orally administered delta-9-THC has shown a significant IOP lowering effect, but this effect is short-timed and subject to tolerance phenomena [78]. Patients treated with oral delta-9-THC experience an IOP decrease that peaked 2 h after administration and then tended to dissipate in 4 h. This temporary effect would require several drug administrations and would lead to toxicity phenomena. Another problem to be addressed is tolerance. From that, perspective oral cannabinoids are unlikely to become a feasible glaucoma treatment option. On the other hand, oral PEA supplementation has shown a good IOP lowering effect (IOP mean reduction of 3.5 ± 1.2 mmHg after two months of treatment) together with good tolerability. This molecule may therefore be a useful tool in glaucoma management, but further studies are needed to better clarify its efficacy and safety [79].

Intravenous administration of delta-9-THC demonstrated IOP lowering capabilities, but the IOP decrease was short-timed and side effects were frequent and severe (pre-syncopal symptoms registered in 30% of study population). Therefore, intravenous administration of delta-9-THC should be considered for research purposes only, rather than as a concrete therapeutic option.

The IOP lowering effects of delta-9-THC derived eye drops are still controversial. While some studies demonstrated no significant IOP reduction in subjects administered topical delta-9-THC, the study from Merritt et al. suggests possible IOP lowering effects of this compound. The IOP decrease described by Merritt was nevertheless modest and short-lived. Therefore, the utility of delta-9-THC derived eye drops in glaucoma seems questionable [13]. A similar brief IOP lowering effect was also demonstrated for a synthetic cannabinoid receptor agonist called WIN55212-2 [80]. The IOP decrease registered for this compound was significant, but again the very short-lasting effects (2 h) makes these eye drops unlikely to be considered a feasible treatment option. Canasol, a delta-9-THC derived eye drops commercially sold in Jamaica, in spite of its popularity among glaucoma patients, it still lacks proven efficacy and safety evidence [72].

Finally, marijuana smoking has been proposed as a possible treatment option for glaucoma patients. Inhaled delta-9-THC via marijuana smoking has demonstrated significant IOP lowering capabilities in all conducted studies [4,20,56,57,65]. In spite of these excellent results, the short-lasting effect on IOP, the development of tolerance, and the potential damage to the general health advise against the use of marijuana for the treatment of glaucoma [81,82,83]. Moreover, the effects of prolonged cannabinoids’ usage on general health and neurocognitive processes represent a main issue [84]. The vast majority of manuscripts included in this review only investigated short-time side effects of cannabinoids while the effects of prolonged usage of these molecules have not been disclosed. Warnings for potential damages to general health are otherwise confirmed by dedicated literature [85,86,87,88]. Prolonged cannabinoids’ usage, especially if inhaled, may be connected with impaired visual short-term memory, reduced effectiveness in visual processing [89], intermitted light phenomena [90,91], paranoia, hallucinations, mental health co-morbidities, and impaired memory [69,70,71,72]. Furthermore, no data is available for drug-drug and drug-vehicle interactions [21,92]. This lack of knowledge may lead to serious and unexpected side effects as well as to dangerous therapeutic failures.

### Future Directions

Some crucial issues such as systemic side effects and tolerance development advise against cannabinoids usage as IOP lowering drugs. Beside these observations, we must notice that in recent years a huge number of efficient and safe IOP lowering drugs have been delivered to market and new anti-glaucoma agents such as small interfering RNA (siRNA) and Adenosine receptors agonists are available in the pipeline [93]. In this scenario, clinical application of cannabinoids as IOP lowering agents seems even more unlikely. However, cannabinoids may be exploited in glaucoma therapy thanks to their neuroprotective capabilities [11]. The crescent evidences a pathogenetic role of neurodegeneration in glaucoma bring research towards the identification of safe and efficient neuroprotective agents. In this specific field, cannabinoids may therefore play a main role thanks to their neuroprotective, vasorelaxant, and antioxidant properties [11,75]. Cannabinoids have demonstrated the inhibition of Glutamate and Endothelin-1 release to reduce RGC death and to inhibit production of nitric oxide and inflammatory cytokines that are responsible for oxidative stress [14,15,17,18]. All these properties may play an important role against neurodegeneration in glaucoma patients. Moreover, some specific molecules such as the synthetic cannabinoid HU-210 demonstrated excellent neuroprotective capabilities without any psychotropic effect [16]. The search for new molecules with a neuroprotective role but without systemic side effects may therefore be the key for a clinical application of cannabinoids in glaucoma. Besides neuroprotection, cannabinoids may otherwise be investigated also in age-related macular degeneration. Actually, cannabinoids showed to inhibit angiogenesis by decreasing vascular endothelial growth factor expression, the main actor of the choroidal neovascularization process. In conclusion, cannabinoids neuroprotective capabilities still need to be completely understood in order to exploit these promising molecules.

## 4. Material and Methods

**Figure 5:**
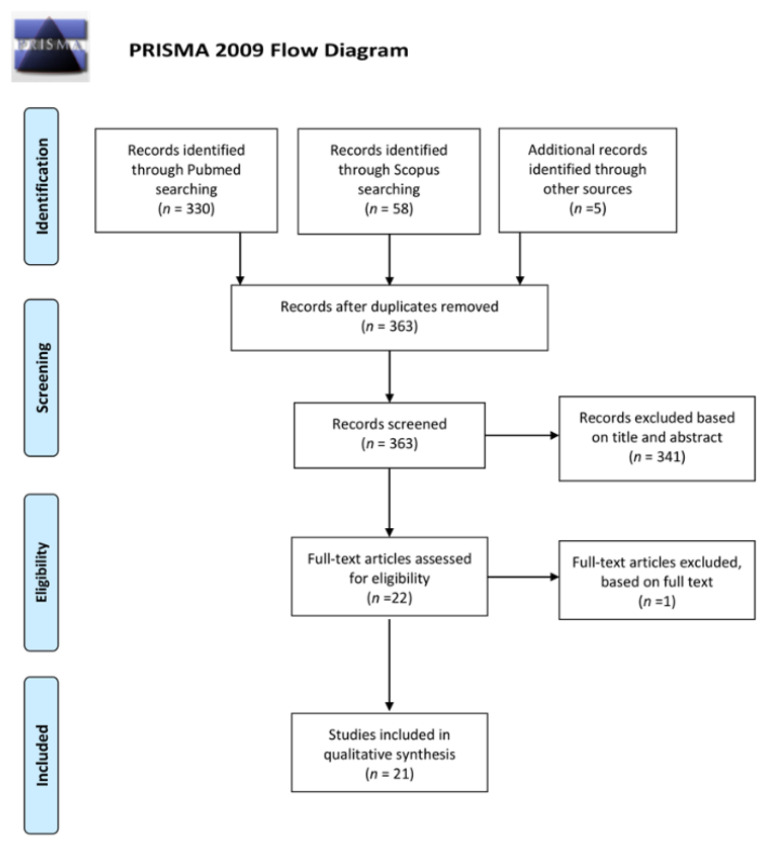
Prisma flowchart.

The review was performed according to the PRISMA guidelines (Preferred Reporting Items for Systematic Reviews and Meta-Analyses) [94]. Two investigators (A.P. and L.P.) independently search studies indexed on Pubmed and Scopus during March 2020, therefore only studies published before 1 March 2020 are cited in this review. The following combinations of keywords: glaucoma/ocular hypertension/glaucomatous disease AND cannabinoids, cannabis, canna*, Tetrahydrocannabinol, Cannabidiol, and Cannabinol have been used for the research. Articles not written in English, narrative reviews, animal model studies, case reports, non-original studies, and studies not subjected to a peer-review were excluded. The search workflow was designed in adherence to the preferred reporting items for systematic reviews and meta-analyses (PRISMA) statement [94] (Figure 5).

All identified articles were independently evaluated, in terms of their titles and abstracts, by two reviewers (A.P. and L.P.) to identify relevant articles. In addition, the references of identified articles were manually checked to find any potential studies relevant for review purposes. The same reviewers selected the studies according to the inclusion and exclusion criteria. Any disagreement was assessed by consensus and a third reviewer (M.F) was consulted when necessary. For unpublished data, no effort was made to contact the corresponding authors. After removing duplicates out of the 363 records identified 341 were excluded on the basis of title and abstract. The full texts of the remaining 22 were assessed for eligibility: 1 record was excluded based on full text and 21 were included for the analysis [4,20,42,56,57,58,59,60,61,62,63,64,65,66,67,68,69,70,71,73,74]. All the selected records were evaluated to define the strength of evidence, according to the Oxford Centre for Evidence-Based Medicine (OCEM) 2011 guidelines and the Scottish Intercollegiate Guideline Network (SIGN) assessment system for individual studies, as implemented in the Preferred Practice Patterns of the American Academy of Ophthalmology [95,96]. Finally, the quality of evidence was also assessed, based on the Grading of Recommendations Assessment, Development, and Evaluation (GRADE) system [97]. The included studies were listed in the Supplementary Materials (Tables S1–S4). Five randomized clinical trials, seven case-control studies, three prospective studies, and 6 case series. The IOP lowering effect and of orally administered, inhaled, topical and intravenous cannabinoids were investigated and related adverse events. The present systematic review presents a qualitative analysis of available data in a narrative fashion due to the heterogeneity and the design of the studies; moreover, the majority of the analyzed papers referred to the 70s and 80s.

## 5. Conclusions

The published evidence suggests that the effects of cannabinoids on intraocular pressure are short-timed and affected by tolerance development. Cannabinoids usage may otherwise be harmful for general health and neurocognitive processes therefore their application as anti-glaucoma treatment may be hazardous. Moreover, safe and effective IOP lowering drugs are already available on the market and new promising molecules are at end-stage experimentation. In this setting cannabinoids’ usage as IOP lowering agents seems unlikely. Some cannabinoids otherwise present excellent neuroprotective effects without systemic or psychotropic involvement. These molecules may therefore be exploited in glaucoma treatment thanks to their neuroprotective, vasorelaxant, and antioxidant properties. Further studies on larger populations with longer follow-up time would be necessary to better clarify the neuroprotective capabilities of these compounds as well as their long-time effects on general health.

### Limitations

Despite the large number of manuscripts about cannabinoid usage in glaucoma, the overall scientific evidence on this topic remains controversial. The vast majority of manuscripts included in this review are case series or small clinical studies without a randomization process or a control group. Besides the lack of randomized controlled trials, we must also underline that the majority of cited manuscripts date back to the 70s and early 80s. This may represent an additional limitation because manuscripts of that period often present issues in data disclosure, statistical analysis, population homogeneity, and safety assessment. For these reasons, scientific evidence on this topic remains limited.

## Figures and Tables

**Table 1:** Evidences of potential neuroprotective effects of cannabinoids.

Authors	Study Population	Intervention	Results
Plange et al., 2007 [42]	8 healthy subjects	Single dose of 7.5 mg oral Dronabinol (Delta-9-THC)	Significant decrease in artero-venous retinal passage time
Hommer et al., 2020 [43]	24 healthy subjects	5 mg oral Dronabinol (Delta-9-THC)	Increased optic nerve head blood flow
Green et al., 1978 [44]	Rabbits	Intravenous THC	Increased ocular blood flow (choroidal, iris and ciliary processes flow)
Yoles et al., 1996 [45]	Rats	Intraperitoneal Dexanabinol (HU-211) or vehicle alone	Reduction in electrophysiological and metabolic deficits after optic nerve injury in HU-211 group
Zalish and Lavie, 2003 [46]	18 rats	Intraperitoneal HU-211 or vehicle alone	Observation of unmyelinated and thinly myelinated axons 30 days after optic nerve injury in the treated group compared with controls
Opere et al., 2006 [47]	Isolated bovine retina	Anandamide, ACEA, Methanandamide, WIN55212-2 (superfusion method)	Inhibition of K^+^-induced aspartate retinal release induced by ischemia
Crandall et al., 2007 [48]	14 rats	20-week treatment of intraperitoneal THC or ethanol solution vehicle once weekly	Reduction in GCL loss after episcleral vessel cauterization induced glaucoma in the treated group
Nucci et al., 2007 [49]	Rats	Single systemic administration of URB597 or intravitreal methanandamide alone or in combination with SR141716 or capsazepine	URB597 and methanandamide alone induced a reduction in GCL loss after acute rise IOP-induced ischemia
Pinar-Sueiro et al., 2013 [50]	27 rats	Topical administration of 1% WIN55212-2 or WIN55212-2 1% and AM251 1%, or vehicle alone for two days	Reduction in GCL loss after acute rise IOP-induced ischemia in the WIN55212-2 1% group
Liu et al., 2014 [51]	18 rats	Four-week treatment with intravitreal HU-211 1 mg or saline solution	Lower reduction in RGC density in the HU-211-group after episcleral vessel cauterization induced glaucoma

HU-211: Dexanabinol, THC: Tetrahydrocannabinol.

**Table 2:** Intra Ocular Pressure (IOP) lowering effects of orally administered cannabinoids.

Authors	Study Population	Intervention	Results
Hepler et al., 1976 [56]	120 subjects affected by POAG	single dose of 5 mg delta-9-THC	14% and 15% IOP lowering after 30 and 180 min from administration
		single dose of 10 mg delta-9-THC	23% and 18% IOP lowering after 30 and 180 min from administration
		single dose of 20 mg delta-9-THC	24% and 23% IOP lowering after 30 and 180 min from administration
Hepler et al., 1976 [57]	40 healthy subjects	single dose of 5 mg synthetic delta-9-THC	10% IOP lowering at 30 min after administration
		single dose of 10 mg synthetic delta-9-THC	10% IOP lowering at 30 min after administration
		single dose of 20 mg synthetic delta-9-THC	16% IOP lowering at 30 min after administration
Newell et al., 1979 [58]	18 subjects affected by POAG or ocular hypertension	single dose of Nabilone 0.5 mg	27.9% IOP lowering between two to four hours after administration
Tiedeman et al., 1981 [59]	15 subjects affected by ocular hypertension	5 mg BW29Y (5 subjects)	no significant effects on IOP
		10 mg BW29Y (10 subjects)	no significant effects on IOP
	22 subjects affected by ocular hypertension	4 mg BW146 (9 subjects)	23% IOP lowering
		8 mg BW146 (10 subjects)	25% IOP lowering
		12 mg BW146 (3 subjects)	42% IOP lowering
Flach AJ 2002 [60]	9 patients affected by POAG on maximally tolerated medical therapy	10 mg to 80 mg oral delta-9-THC daily (in addition to regular glaucoma therapy). Treatment duration between 3 and 36 weeks.	initial IOP lowering limited by tolerance development (mean IOP reduction not disclosed)
Tomida et al., 2006 [61]	6 subjects affected by POAG or ocular hypertension	single dose of 5 mg delta-9-THC	14% and 5.3% IOP lowering at 2 and 4 h from administration
		single dose of 20 mg cannabidiol	no significant effects on IOP
		single dose of 40 mg cannabidiol	no significant effects on IOP
Plange et al., 2007 [42]	8 healthy subjects	single dose of 7.5 mg Dronabinol	10% IOP lowering two hours after administration
Gagliano et al., 2011 [62]	42 subjects affected by POAG or ocular hypertension	300 mg palmitoyl-ethanolamide twice a day for two months (in addition to timolol 0.5%)	16.2% IOP lowering after two months of treatment
Pescosolido et al., 2011 [63]	15 subjects undergoing prophylactic iridotomy	300 mg palmitoyl-ethanolamide twice a day for 15 days before iridotomy	24%, 28% and 31% IOP lowering at 15, 30 and 120 min after iridotomy (vs. placebo)
Strobbe et al., 2013 [64]	40 subjects affected by ocular hypertension	300 mg palmitoyl-ethanolamide twice a day for 3 months	3.7% IOP lowering

POAG: primary open angle glaucoma, IOP: intraocular pressure, THC: Tetrahydrocannabinol.

**Table 3:** IOP lowering effects of inhaled cannabinoids.

Authors	Study Population	Intervention	Results
Hepler et al., 1971 [4]	11 healthy subjects	18 mg delta-9-THC	24% IOP lowering (limited data available)
Flom et al., 1975 [20]	15 healthy subjects	12 mg delta-9-THC	13% IOP lowering after 80 min from administration
Hepler et al., 1976 [56]	136 subjects affected by POAG administered with 1, 2, or 4% THC cigarette	20 mg delta-9-THC (2 g marijuana cigarette with 1% delta-9-THC)	29% and 22% IOP lowering after 30 and 180 min from administration
		40 mg delta-9-THC (2 g marijuana cigarette with 2% delta-9-THC)	25% and 17% IOP lowering after 30 and 180 min from administration
		80 mg delta-9-THC (2 g marijuana cigarette with 4% delta-9-THC)	34% and 22% IOP lowering after 30 and 180 min from administration
Hepler et al., 1976 [57]	40 healthy subjects	1% natural delta-9-THC cigarette	30% IOP lowering 30 min after administration
		2% natural delta-9-THC cigarette	25% IOP lowering 30 min after administration
		4% natural delta-9-THC cigarette	34% IOP lowering min after administration
		1% synthetic delta-9-THC cigarette	15% IOP lowering 30 min after administration
		2% synthetic delta-9-THC cigarette	23% IOP lowering 30 min after administration
		4% synthetic delta-9-THC cigarette	24% IOP lowering 30 min after administration
Merritt et al., 1980 [65]	18 glaucoma patients (12 affected by POAG, 6 affected by secondary glaucoma)	18 mg delta-9-THC (0.9 g marijuana cigarette with 2% delta-9-THC)	14.5% and 23.4% IOP lowering after 30 and 90 min from administration

(POAG: primary open angle glaucoma, IOP intraocular pressure, THC Tetrahydrocannabinol.).

**Table 4:** IOP lowering effects of topical cannabinoids.

Authors	Study Population	Intervention	Results
Merritt et al., 1981 [67]	6 hypertensive glaucoma patients	single 0.05% delta-9-THC eye drop	No significant IOP lowering
		single 0.1% delta-9-THC eye drop	No significant IOP lowering
Merritt et al., 1981 [68]	8 hypertensive glaucoma patients	single 0.01% delta-9-THC eye drop	No significant IOP lowering
		single 0.05% delta-9-THC eye drop	No significant IOP lowering
		single 0.1% delta-9-THC eye drop	No significant IOP lowering
Green et al., 1982 [69]	16 healthy subjects	single administration of 1% delta-9-THC eye drop	No significant IOP lowering
Jay et al., 1983 [70]	28 healthy subjects	1% delta-9-THC eye drop four times a day for a week	No significant IOP lowering
Porcella et al., 2001 [71]	8 glaucoma patients (4 POAG, 2 malformative glaucoma, 1 pigmentary glaucoma, 1 angle closure glaucoma)	25 µg of WIN55212-2, two drops	15 ± 0.5% IOP lowering after 30 min from administration
		50 µg of WIN55212-2, two drops	23 ± 0.9% IOP lowering after 30 min from administration
Pescosolido et al., 2018 [72]	5 glaucoma patients (4 POAG and 1 post-uveitic glaucoma)	Bediol (containing 3–6 mg/mL of delta-9-THC and 4–8 mg/mL of cannabidiol) twice a day for 30 days and then switched to topical Bedrocan (containing 18–23 mg/mL of delta-9-THC and 1.2–18 mg/mL of cannabidiol) twice a day for 30 days	No significant IOP lowering regardless of employed compound.

POAG: primary open angle glaucoma, IOP: intraocular pressure, THC: Tetrahydrocannabinol.

**Table 5:** IOP lowering effects of intravenous cannabinoids.

Authors	Patient Population	Intervention	Results
Purnell et al., 1975 [74]	2 healthy subjects	Single dose of 6.7 mg THC in subject 1	62% IOP lowering 90 min after administration
		Single dose of 3.0 mg THC in subject 2	42% IOP lowering 30 min after administration
Cooler et al., 1976 [75]	10 healthy subjects	0.022 mg/Kg delta-9-THC	29% IOP lowering
		0.044 mg/Kg delta-9-THC	37% IOP lowering

IOP: intraocular pressure, THC: Tetrahydrocannabinol.

## Supplementary Materials

The following tables are available online at https://www.mdpi.com/2077-0383/9/12/3978/s1, Table S1, Orally administered cannabinoids evidences; Table S2, Inhaled cannabinoids evidences; Table S3, Topical cannabinoids evidences; Table S4, Intravenous cannabinoids evidences.
