# Supplementary material for: Cannabinoids in Glaucoma Patients: The Never-Ending Story
Source: J Clin Med. 2020 Dec 8;9(12):3978. doi: 10.3390/jcm9123978 (PMC7763320; doi:10.3390/jcm9123978)
Supplement: Supplementary file 1 [file jcm-09-03978-s001.pdf]

Table S1. Orally administered cannabinoids evidences.

Authors	Year	Study Design	Sample	Intervention	IOP lowering	Level	Strength	Grade
Hepler RS	1976	Case-Control Study	120	5-10-20 mg delta-9-THC	14-15%	4	IV	Low
Hepler RS	1976	Case-Control Study	40	5-10-20 mg delta-9-THC	23-18%	4	IV	Low
Newell FW	1979	Prospective Study	18	Nabilone 0.5 mg	24-23%	4	IV	Low
Tiedeman JS	1981	Randomized clinical trial	37	5-10 mg BW29Y 4-8-12 mg BW146	10%	2	II	Moderate
Flach AJ	2002	Prospective study	9	10 mg to 80 mg oral delta-9-THC	10%	4	IV	Very Low
Tomida I	2006	Randomized clinical trial	6	5 mg delta-9-THC, 20-40 mg cannabidiol	16%	2	II	Moderate
Plange N	2007	Case series	8	7.5 mg Dronabinol	27.9%	4	IV	Very Low
Gagliano C	2011	Randomized clinical trial	42	300 mg palmitoyl-ethanolamide	no significant effects on IOP	2	II	Moderate
Pescosolido N	2011	Prospective Study	15	300 mg palmitoyl-ethanolamide	no significant effects on IOP	4	IV	Low
Strobbe E	2013	Randomized clinical trial	40	300 mg palmitoyl-ethanolamide	23%	2	II	Moderate

POAG: primary open angle glaucoma, IOP intraocular pressure, THC Tetrahydrocannabinol.

Table S2. Inhaled cannabinoids evidences.

Authors	Year	Study Design	Sample	Intervention	IOP lowering	Level	Strength	Grade
Hepler RS	1971	Case series	11	18 mg delta-9-THC cigarette	24% IOP lowering	4	IV	Very Low
Flom MC	1975	Case-Control Study	15	12 mg delta-9-THC cigarette	13% IOP lowering	4	IV	Low
Hepler RS	1976	Case-Control Study	136	20-40-80 mg delta-9-THC cigarette	17%-34% IOP lowering	4	IV	Low
Hepler RS	1976	Case-Control Study	40	1-2-4% natural vs synthetic delta-9-THC cigarette	15%-34% IOP lowering	4	IV	Low
Merritt JC	1980	Case series	18	18 mg delta-9-THC cigarette	14.5%- 23.4% IOP lowering	4	IV	Low

POAG: primary open angle glaucoma, IOP intraocular pressure, THC Tetrahydrocannabinol.

Table S3. Topical cannabinoids evidences.

Authors	Year	Study Design	Sample	Intervention	IOP lowering	Level	Strength	Grade
Merritt JC	1981	Randomized clinical trial	6	0.05-0.1% delta-9-THC eye drop	No significant IOP lowering	2	II	Moderate
Merritt JC	1981	Case series	8	0.01-0.05-0.1% delta-9-THC eye drop	No significant IOP lowering	4	IV	Very Low
Green K	1982	Case-control study	16	1% delta-9-THC eye drop (single administration)	No significant IOP lowering	4	IV	Low
Jay WM	1983	Case-control study	28	1% delta-9-THC eye drop (four times a day)	15±0.5% IOP lowering	4	IV	Low
Porcella A	2001	Case-control study	8	25-50 μg of WIN55212-2 eye drop	23±0.9% IOP lowering	4	IV	Very Low
Pescosolido N	2018	Case series	5	Bediol and topical Bedrocan eye drop	No significant IOP lowering	4	IV	Very Low

POAG: primary open angle glaucoma, IOP: intraocular pressure, THC: Tetrahydrocannabinol.

Table S4. Intravenous cannabinoids evidences.

Authors	Year	Study Design	Sample	Intervention	IOP lowering	Level	Strength	Grade
Purnell WD	1975	Case series	2	3.0-6.7 mg THC	42-62% IOP lowering	4	IV	Very Low
Cooler P	1976	Case-control study	10	0.022-0.044 mg/Kg delta-9-THC	29-37% IOP lowering	4	IV	Very Low

IOP: intraocular pressure, THC: Tetrahydrocannabinol.
